# A Cohort Study of Emergency Surgery Caseload and Regional Anesthesia Provision at a Tertiary UK Hospital During the Initial COVID-19 Pandemic

**DOI:** 10.7759/cureus.8781

**Published:** 2020-06-23

**Authors:** Stuart Wade, Ganeshkrishna Nair, Hafis A Ayeni, Amit Pawa

**Affiliations:** 1 Anaesthesia, Guy's and St Thomas' NHS Foundation Trust, London, GBR; 2 Anaesthesia, Guy’s and St Thomas' NHS Foundation Trust, London, GBR

**Keywords:** covid-19, regional anesthesia, emergency surgery, personal protective equipment

## Abstract

Study objective

Analysis of emergency cases performed during initial coronavirus disease 2019 (COVID-19) pandemic and the proportion completed under regional anesthesia (RA).

Design

Cohort study comparing surgical caseload during initial seven-week COVID-19 pandemic in 2020. Comparison was made with pre-COVID-19 caseload over the corresponding seven-week timeframe in 2019.

Setting

The setting of the study was emergency surgery theaters at Guy’s and St Thomas’ NHS Foundation Trust, London, UK.

Patients

All patients requiring emergency surgery over the defined study period were reviewed with the exception of obstetric and pediatric populations.

Interventions

Surgical caseload for 2020 and 2019 cohorts established using the Galaxy IT system used to log all operations. All relevant anesthetic charts for the 2020 cohort were subsequently reviewed to ascertain perioperative use of RA.

Measurements

The type of block, mode of approach, experience of the operator, personal protective equipment (PPE) worn, block complications, type of sedation and complications were entered into database.

Main results

A total of 338 emergency surgical cases were performed during the COVID-19 pandemic in 2020, compared to 603 cases over the corresponding period in 2019. This showed a 44% decrease in emergency surgical workload. There was a marked disparity in reduction of surgical caseload by surgical subspecialty. Trauma (137 vs 66 cases), a 52% decrease, and general surgery (193 vs 64 cases), a 66% decrease, were the most pronounced, and explanations for this are explored. RA was performed in 34% (26% as primary technique) of cases during the COVID-19 pandemic. The use of RA as the primary anesthesia technique was noticeably higher than previous UK data (11%), and was prominent in specialties such as general surgery, gynecology and urology, not traditionally completed under RA.

Conclusions

Surgical RA (and general anesthesia avoidance) has a significant role in the future to ensure high-quality perioperative care for patients whilst minimizing exposure to staff and utilization of scarce resources (PPE).

## Introduction

An outbreak of severe pneumonia in Wuhan, Hubei province, China was first reported in December 2019 [1]. The causative agent was identified as a betacoronavirus severe acute respiratory syndrome coronavirus 2 (SARS-CoV-2), a highly infectious organism that predominantly affects the lower respiratory tract in humans. It was subsequently renamed coronavirus disease 2019 (COVID-19) by the World Health Organization (WHO). On April 10, 2020, the WHO characterized COVID-19 disease as a global pandemic [2]. At the time of writing, COVID-19 had spread to 215 countries with over four million confirmed cases and 300,000 deaths worldwide [3].

Initial reports suggested that of those infected up to 20% developed severe disease that required hospitalization with approximately 25% of hospitalized patients needing intensive care unit (ICU) admission. A significant proportion of ICU patients require intubation and ventilation, with numerous associated risks [4-7]. COVID-19 patients will already have marked physiological derangement, and tracheal intubation should therefore only be attempted when clinically mandated [6,7]. 

In the UK, National Health Service (NHS) trusts were ordered to reconfigure services and personnel to free up capacity for the predicted rise in ICU admissions and ventilated patients [8]. The provision of core services such as obstetrics, emergency surgery and cancer care needed to be maintained whilst keeping patients and staff safe.

It is known that high-risk aerosol-generating procedures (AGP) such as tracheal intubation may put the anesthesiologists and theater team at risk of nosocomial infections [9,10]. It is therefore imperative that the appropriate level of personal protective equipment (PPE) is available to all staff exposed to a potential AGP with baseline protection being fluid-resistant gown, gloves, visor or face-shield and respiratory protection. Filtering facepiece respirators (N95/FFP3) are currently the minimum standard, but ideally powered air-purifying respirators (PAPRs) should be available [7,9]. 

Regional anesthesia (RA) affords many obvious solutions to the unique issues presented by COVID-19 patients. The patient would receive safe effective anesthesia with superior analgesia and a lower risk of postoperative complications [11,12]. The avoidance of a general anesthesia (GA) is especially relevant for patients who are currently COVID-19 positive but who also require emergency surgery and the loss of respiratory reserve this might precipitate. Use of a RA technique could also reduce the risk of exposing theater staff to an AGP, spare the need for anesthetic drugs that may be required in the ICU and reduce the need for FFP3 mask usage if the surgery was not aerosol generating [12]. 

Guy's and St Thomas’ NHS Foundation Trust’s (GSTT) position in South London placed it at the epicenter of the COVID-19 pandemic in the UK. A large proportion of surgery is routinely completed under RA and a majority of the anesthesiologists are proficient in its provision. We report on the cases performed at GSTT during the early stage of the COVID-19 pandemic in 2020, with a direct comparison of the emergency caseload performed over the same timeframe in 2019. We have explored how the emergency surgery case mix changed significantly during the COVID-19 pandemic and how the role of RA can be expanded, as provision of anesthesia for patients with COVID-19 becomes the new normality.

## Materials and methods

Institutional approval was obtained from the local clinical governance department to undertake this service evaluation. The case series consisted of a snapshot search of all operating theater activity at GSTT between March 23, 2020 and May 10, 2020 on the Galaxy computer system, the IT system used to log all operations in our trust. 

Scanned operating notes and anesthetic charts for all emergency procedures within the study timeline were reviewed, with the exception of obstetric and pediatric cases. The type of block, mode of approach (landmark, peripheral nerve stimulator and or ultrasound), experience of the operator, PPE worn, block complications, type of sedation (if used) and complications (e.g. conversion to GA) were noted. Duplicate entries were removed as were those with inadequate documentation. Anonymized data were then entered into a secure database for analysis. 

The cases performed in the emergency theaters of GSTT during the corresponding period from 2019 were also identified. Paper-based searches were not employed due to current COVID-19 restrictions in getting access to paper notes.

## Results

A total of 338 urgent or emergency cases performed in main theaters at GSTT over a period of seven weeks during the COVID-19 pandemic were analyzed. During the corresponding period last year, 603 cases were performed. The number of surgeries performed by the various specialties is illustrated in Table 1.

**Table 1:** Surgeries performed as per specialty

Specialty	2019	2020
General surgery	193 (32%)	64 (19%)
Gynecology	58 (9.6%)	51 (15.1%)
Plastic surgery	43 (7.1%)	40 (11.8%)
Thoracic	17 (2.8%)	21 (6.2%)
Trauma and orthopedics	137 (22.7%)	66 (19.5%)
Urology	48 (8%)	42 (12.4%)
Vascular	61(10.1%)	40 (11.8%)
Miscellaneous	14 (2.3%)	14 (4.1%)
Transplant	32 (5.3%)	0
Total	603	338

**Figure 1:**
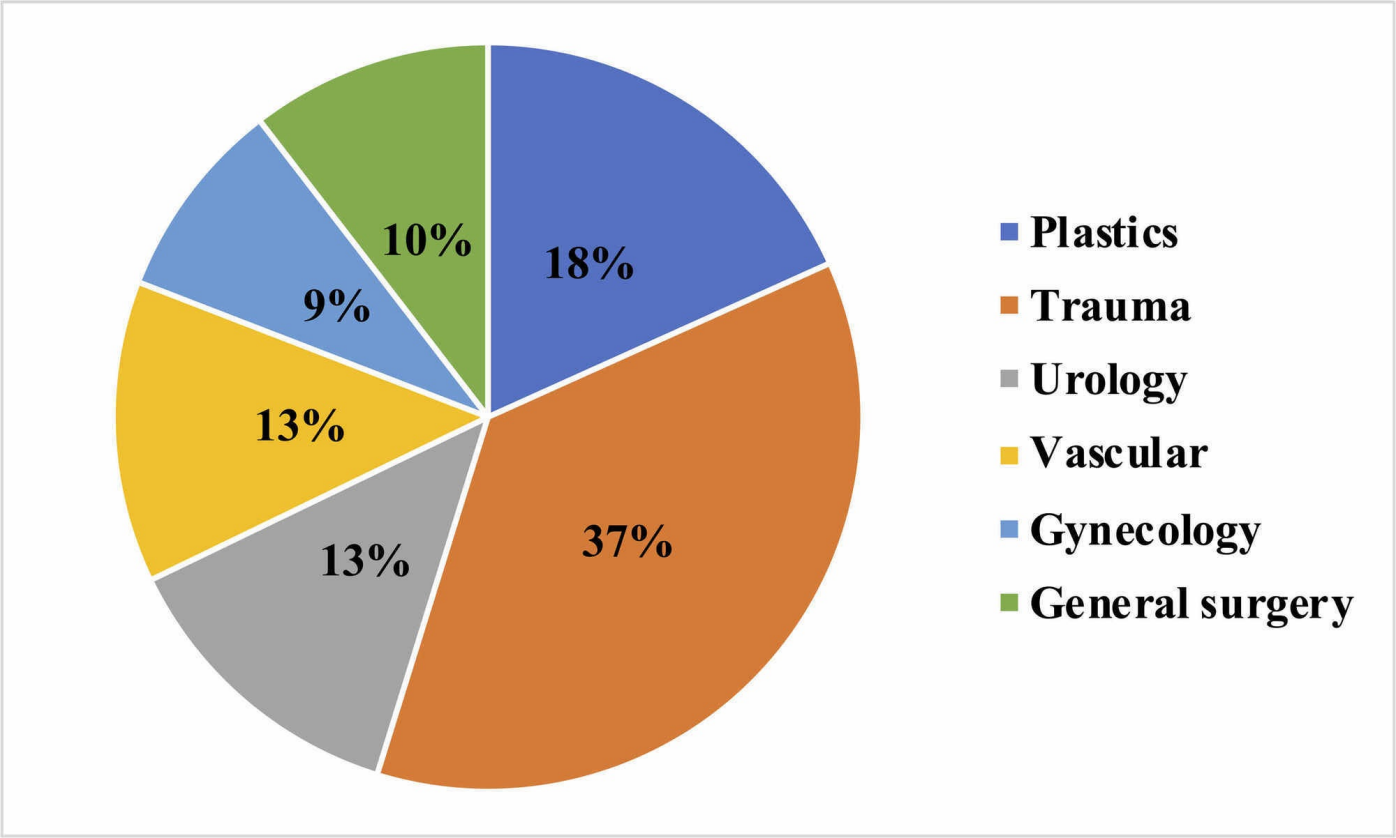
Chart showing various specialties for which regional anesthesia techniques were used

RA was performed for 115 (34%) of these cases. Our trust policy was to swab all patients but still treat them as suspected COVID positive. All RA procedures were performed in theater. The age of patients ranged from 17 to 98 years. Of these patients, 53 were male and 62 were female. RA was performed by consultant anesthesiologists in 78 of these cases, and 37 by senior registrars. The surgical specialties and types of surgeries for which RA techniques were used are summarized in Figure 1.

A total of 125 RA procedures were performed for 115 surgeries; multiple RA techniques were used for 10 operations. The specialties which had the highest proportion of cases completed with the use of RA were trauma and orthopedics (62.1%) and plastic surgery (52.5%). The use of RA across all specialties is illustrated in Table 2. 

**Table 2:** Emergency cases undertaken during seven-week period of 2020 and proportion of cases using RA RA, regional anesthesia; GA, general anesthesia

Specialty	All cases	RA cases (% of specialty)	RA + GA cases (% of specialty)	All RA cases (% of specialty)
General surgery	64	6 (9.4)	6 (9.4)	12 (18.8)
Gynecology	51	9 (17.6)	1 (2)	10 (19.6)
Plastic surgery	40	19 (47.5)	2 (5)	21 (52.5)
Thoracic	21	0	0	0
Trauma and orthopedics	66	27 (40.9)	14 (21.2)	41 (62.1)
Urology	42	15 (35.7)	1 (2.4)	16 (38.1)
Vascular	40	13 (32.5)	2 (5)	15 (37.5)
Miscellaneous	14	0	0	0
Total	338	89	26	115

RA was used as the sole anesthetic technique for 89 surgeries (Table 3), while RA was used as an analgesic technique for 26 surgeries (Table 4). The RA techniques used are summarized in Table 5. All procedures were performed under aseptic conditions, and 11 procedures were documented to have been performed using PPE suitable for AGP. All central neuraxial blocks and two fascia iliaca blocks were performed using the landmark technique, while the remainder were performed using ultrasound guidance. 

**Table 3:** List of surgical procedures for which regional anesthesia was used as the sole anesthetic technique

Surgical procedures	
Trauma and orthopedics	
Hip fracture surgery	18
Open reduction internal fixation of fractures	9
Urology	
Trans urethral resection of bladder tumor	3
Prostate surgery	3
Cystoscopy + proceed	4
Scrotal surgeries	5
General surgery	
Open appendectomy	1
Incarcerated hernia repair and mesh	1
Rectal/perianal procedures	3
Incision and drainage abdominal wall abscess	1
Vascular	
Lower limb amputations	7
Washout/debridement	3
Arteriovenous fistula surgeries	3
Plastic surgery	
Hand trauma	19
Gynecology	
Mini laparotomy and salpingectomy	1
Hysteroscopy and polypectomy	1
Evacuation of retained products of conception	7

**Table 4:** List of surgical procedures for which regional anesthesia was used as an analgesic technique

Surgical procedures	
Trauma and orthopedics	
Hip fracture surgery	6
Open reduction internal fixation of fractures	5
Washout/removal of metalwork	3
Urology	
Nephrectomy	1
General surgery	
Laparotomy	5
Open cholecystectomy	1
Vascular	
Lower limb amputations	1
Insertion of femoral arterial stent	1
Plastic surgery	
Hand trauma	2
Gynecology	
Bilateral salpingo-oophorectomy	1

**Table 5:** List of regional anesthesia techniques used for surgery

Regional anesthesia techniques used for surgery	
Ankle	3
Adductor canal block	2
Axillary	8
Brachial p lexus block (not specified)	3
Thoracic epidural	3
Erector spinae plane	1
Femoral	4
Fascia iliaca	7
Forearm blocks	4
Infraclavicular	14
Interscalene	2
Popliteal	7
Rectus sheath	1
Spinal	58
Supraclavicular	8
Total	125

No sedation was used in 31 patients, while 47 patients received some form of sedation. One patient (finger flexor sheath washout under axillary block) needed local infiltration by surgeons during the operation, while three patients (flexor tendon repair and removal of foreign body foot under ankle block, above knee amputation under spinal and drainage of scrotum under spinal) required conversion to GA. The patients who needed conversion to GA all had negative COVID swabs in hospital, and were all discharged with no postoperative complications.

## Discussion

The first seven weeks of the COVID-19 pandemic at GSTT resulted in 338 urgent or emergency cases being completed. This compares to 603 cases over the same time period in 2019, which is at least a 44% decrease in expected emergency surgical workload for this time of year. It is worthwhile noting that the true number of cases performed in 2019 were likely to be higher than 603 as these figures do not reflect the emergency cases performed at the end of elective operating lists, and the hand trauma cases performed in the dedicated hand trauma department. It is likely therefore that the true reduction of emergency surgical work is even greater than 44%.

Irrespective of the actual number, the significant decrease in emergency surgical procedures mirrors recent national published UK data [13]. NHS England reported 916,581 people in England attended accident and emergency departments (A&E) in April 2020, 57% down on the April 2019 figure. Furthermore, those admitted to hospital via A&E over the same timeframe were down to 327,000, a 39% decrease from April 2019 [13].

Explanations for this seismic shift in how the public are using health care are multifactorial and include reduced health seeking behavior for fear of contracting COVID-19, and not wishing to be a burden on the NHS during the pandemic [14]. Our data show an overall decrease in surgical workload; however, there is a marked disparity when you analyze cases performed by surgical subspecialty. Some of this disparity may be explained by government guidelines during the pandemic. At GSTT, there was complete cessation of transplant surgery (32 vs 0, 100% decrease) during the COVID-19 due to restructuring of acute services and an understandable reluctance to commence immunosuppressant therapy during the pandemic [8]. Similarly, the decrease seen in trauma workload (137 vs 66, 52% decrease) can in some way be explained by a reduction in sport or work-related injury due to government stay at home guidelines [15]. 

The largest single fall in emergency caseload however was seen in general surgery (193 vs 64, 66% decrease) that along with trauma and orthopedics constituted the major reduction of cases seen at our trust. This observation is not unique, one study covering three major hospitals in northern Italy reported an 86% decrease in surgical emergencies in the month following the lockdown order from the Italian government [16]. Explanation for this phenomenon includes a higher proportion surgical cases being managed conservatively at home by the general practitioner or suboptimal diagnosis in suspected COVID-19 patients, due to the distracting illness or reduced investigation. Local factors such as patients presenting to a local hospital (rather than GSTT) or lack of access to scanned documents by our research team could possibly account for a small variation. The significant reduction we have observed however remains largely unexplained. Anecdotally we are now starting to see an increase in general surgical cases, who have presented later and with more advanced pathology.

During the COVID-19 pandemic, the proportion of cases in the non-obstetric population where RA was performed was 34% (26% as primary technique). Additionally, we were unable to collate data on much of the cancer work that was transferred to the partnered independent sector with a high proportion of urology cases, also performed under RA. Estimation of the proportion of cases performed under RA prior to COVID-19 varies with the Fifth National Audit Project (NAP5) showing 11% of non-obstetric procedures were performed under RA +/- sedation [17,18]. Our study concurs with other expert opinion that a higher proportion of cases are now tending to be performed under RA due to the pressures of the COVID-19 pandemic [18]. A weakness of this analysis is the fact that extracting the true number of cases performed under RA at our institution during the corresponding period in 2019 was not possible. Inadequate capture within the Galaxy system in 2019 when recording mode of anesthesia, along with an insufficient number of scanned anesthetic charts from the emergency theaters, ensured any comparator figure would be incomplete and therefore inaccurate.

The analysis has also showed some noticeable changes in the anesthetic practice of which operations were completed with RA +/- sedation as the primary mode of anesthesia. Examples from this study include the operations evacuation retained products conception (ERPC), cystoscopy/ureteric stent, open appendectomy and mini laparotomy/salpingectomy. These procedures would previously have been performed under GA unless contraindicated. Anecdotally this has led to a "change in surgical mindset" whereby more cases have been performed under RA as the pandemic has progressed as both awareness and confidence in the techniques have grown. This is especially evident in the surgical specialties of gynecology, urology and general surgery where RA has not traditionally been the primary anesthetic of choice.

Much of this change in practice has been initiated during the team huddle which takes place before commencement of the operating list, during which time the surgeon, anesthesiologist and theater team will discuss how to best proceed with the case. In addition to the usual WHO pre-list team brief, the discussion will include: (1) Could this case be safely performed under RA? (2) How can we reduce AGP/keep staff exposure to a minimum? (3) What level of PPE is required? As with any team and anesthetic brief, all eventualities should be covered including planning for a failed block and conversion to a GA. During this study (3/89, 3.3%), patients required conversion to GA which is just above the Royal College of Anesthesiologists (RCoA) guidance of <3% for emergency surgery [19].

The PPE guidance for our trust during the COVID-19 pandemic was that all emergency surgical patients should be treated as “COVID-19 suspected”; therefore, the majority of anesthetics performed used PPE for AGP (fluid-resistant gown, gloves, visor and FFP3 respirators or PAPR hoods). The advice for non-AGP is now for standard surgical facemask (contact PPE) with many of regional anesthesiologists comfortable siting blocks using this method [20]. However full respirator FFP3/PAPR hood PPE should still be available for medical personnel at higher risk or simply personal choice [18,20]. Contact PPE provides several institutional benefits, including removal of the 20-minute pre- and post-AGP settling time (theoretically increasing theater productivity), increased staff comfort and reduced supply requirement for FFP3 level PPE. There are also potential patient benefits, including improved communication, shorter waiting times and reduced post anesthetic care requirements [18,20]. Only 11 anesthesiologists during our study fully documented the level of PPE used. This should be addressed in the future by being a routine part of anesthetic documentation in the post-COVID-19 era. 

When consenting patients, anesthesiologists must ensure that the patient is aware of the benefits, material risks and any reasonable alternative or variant treatments [18]. The consent process should be undertaken by the responsible anesthesiologist, prior to the patient arriving in theater [21]. During the COVID-19 pandemic, competing demands of reducing staff exposure whilst minimizing the use of a scarce resource (PPE) resulted in (anecdotally) much of the consent process taking place in the operating theater. As PPE stocks are replenished and the role of RA continues to expand in the post COVID-19 era, anesthesiologists should again prioritize full informed consent giving the patient time to make a fully educated choice for their future care [21].

As the number of new COVID-19 cases reduce and as this phase of the pandemic comes under control, it is likely that for some time to come perioperative care will look very different from pre-COVID practice. We will be faced with a large backlog of cases requiring surgery and increasing time pressures. It is plausible that surgical RA (and GA avoidance) will play a significant role to ensure high-quality perioperative care for specific procedures and patient groups. It is imperative therefore to ensure that adequately experienced practitioners are available to perform this, and that education and training in RA is delivered and maintained. It may be prudent to use this opportunity to establish RA block rooms or mobile block teams to facilitate this process and ensure that patients have equitable access to RA.

## Conclusions

The COVID-19 pandemic was associated with a significant decrease in the number of emergency operations performed when compared to the corresponding period in the previous year at a major NHS trust. The most pronounced reduction in caseload was in the subspecialties of general surgery and trauma and orthopedics. RA was increasingly used as the sole mode of anesthesia for patients during the COVID-19 pandemic. The advantages of RA during the COVID-19 pandemic included improved perioperative care, minimizing exposure of the pathogen to patients and staff and less utilization of scarce resources (PPE).
